# A New Role of NAP1L1 in Megakaryocytes and Human Platelets

**DOI:** 10.3390/ijms232314694

**Published:** 2022-11-24

**Authors:** Martin Freitag, Hansjörg Schwertz

**Affiliations:** 1 Department of Cardiac Surgery, Heart Center Leipzig-University Hospital, 04289 Leipzig, Germany; 2 Molecular Medicine Program, University of Utah, Salt Lake City, UT 84112, USA; 3 Division of Occupational Medicine, University of Utah, Salt Lake City, UT 84112, USA; 4 Occupational Medicine at Billings Clinic Bozeman, Bozeman, MT 59715, USA

**Keywords:** co-immunoprecipitation, DLAT, ODP2, PDC-E2, megakaryocytes, NAP1L1, overexpression, platelets, protein interactions, sepsis

## Abstract

Platelets (PLTs) are anucleate and considered incapable of nuclear functions. Contrastingly, nuclear proteins were detected in human PLTs. For most of these proteins, it is unclear if nuclear or alternatively assigned functions are performed, a question we wanted to address for nuclear assembly protein 1 like 1 (NAP1L1). Using a wide array of molecular methods, including RNAseq, co-IP, overexpression and functional assays, we explored expression pattern and functionality of NAP1L1 in PLTs, and CD34^+^-derived megakaryocytes (MKs). NAP1L1 is expressed in PLTs and MKs. Co-IP experiments revealed that dihydrolipolylysine-residue acetyltransferase (DLAT encoded protein PDC-E2, ODP2) dynamically interacts with NAP1L1. PDC-E2 is part of the mitochondrial pyruvate-dehydrogenase (PDH) multi-enzyme complex, playing a crucial role in maintaining cellular respiration, and promoting ATP-synthesis via the respiratory chain. Since altered mitochondrial function is a hallmark of infectious syndromes, we analyzed PDH activity in PLTs from septic patients demonstrating increased activity, paralleling NAP1L1 expression levels. MKs PDH activity decreased following an LPS-challenge. Furthermore, overexpression of NAP1L1 significantly altered the ability of MKs to form proplatelet extensions, diminishing thrombopoiesis. These results indicate that NAP1L1 performs in other than nucleosome-assembly functions in PTLs and MKs, binding a key mitochondrial protein as a potential chaperone, and gatekeeper, influencing PDH activity and thrombopoiesis.

## 1. Introduction

Megakaryocytes (MKs) are highly specialized precursor cells, residing in the bone marrow [1] and lungs [2], that produce and finally release platelets (PLTs) [3,4,5]. The development and maturation of MKs and the subsequent production of PLTs by MKs are tightly regulated and depend on external cues [6,7,8]. During the final stages of MK development, the cytoplasm is massively reorganized and long cytoplasmic extensions are formed, coined proplatelets [9]. Two cytoskeletal systems provide the force leading to the shape changes associated with MK maturation and proplatelet formation: actin filaments and microtubules assembled from tubulin subunits [10,11]. More specifically, it was demonstrated that dynein-dependent microtubule sliding acts as the driving force for proplatelet elongation and PLT biogenesis [11]. In this context, it is important to note that such processes are strictly dependent on sufficient availability of ATP as an energy source [12].

Once released into the circulation, PLTs impose as small, and anucleate cells. In the past, PLTs have traditionally been considered simple ‘sacs of glue’ with limited functionality beyond acute hemostasis due to the lack of a cell nucleus. However, in recent years, PLTs have been identified as dynamic effector cells bridging the inflammatory, immune, and hemostatic continuum [6,7,8,13,14,15,16]. One mechanism explaining the multitude of functional responses is that MKs are equipped to sense external stimuli that trigger differential expression and packaging of mRNA and proteins into their progeny, causing distinct PLT functional alterations, especially in disease [6,7,8,17,18,19,20,21]. Hence, although PLTs are anucleate, we found that the PLTs’ array of mRNAs is dynamic and can undergo active re-programming [8,17,18,19]. Furthermore, intricate processes regulate RNA processing, expression, and translational events [22,23,24,25,26,27,28] in PLTs. In concert with maintaining nuclear RNA processing capabilities, numerous studies have shown that PLTs possess nuclear proteins, such as the peroxisome proliferator-activated receptor (PPAR)-c, and retinoid X receptor (RXR) [29,30], and retinoic acid receptor (RAR) [28,31].

Sepsis is a common, and often morbid, syndrome which is characterized by systemic inflammation and dysregulated host immune responses triggered by invading pathogens or toxins (i.e., Toll-like receptor [TLR] agonists like LPS), frequently intensified by injurious agonists generated by the infected organism. The MK-PLT axis plays a major role in orchestrating sepsis-related thromboinflammatory and immunothrombotic events [7,32,33,34,35]. MKs were demonstrated to synergistically react to TLR 2/4 agonists in combination with thrombopoietin (TPO) signaling by increasing their proliferation, and thrombopoiesis [36]. However, this effect could only be observed in immature CD34+ cells, while MKs in the past 7 days of culture demonstrated slightly decreasing numbers when treated with LPS. Finally, PLT aggregation and the formation of heterotypic PLT-leukocyte aggregates in septic patients is associated with increased morbidity and mortality [37,38,39]. Nevertheless, the direct impact of PLTs in the pathobiology of sepsis remains incompletely understood. Furthermore, several cell biology aspects, and mechanistic insight into PLT and MK functions during septic events are still obscure.

The purification and functional characterization of a 53 kDa protein facilitating nucleosome assembly in mammalian cells was first described in 1984 [40]. The NAP-family, including nucleosome assembly protein 1 like 1 (NAP1L1), is a group of proteins with chaperone function, capable of binding histones and coordinating their assemblance into nucleosomal particles [41]. Several additional binding partners for NAP-family members were identified, including transcription factors, proteins regulating nuclear import (karyopherins), and factors involved in cell cycle regulation (reviewed in [41]). Specifically, NAP1L1 was described to promote proliferation of pluripotent stem cells [42] through cell cycle interference. Furthermore, NAP1L1, in concert with NAP1L4, can act as a histone chaperone [43], and serving as a co-factor for ubiquitination client selection [44]. Furthermore, numerous studies describe NAP1L1 as a prognostic marker in select cancer entities (i.e., colon cancer [45], hepatocellular carcinoma [46], ovarian cancer [47], and lung cancer [48]), and its involvement in a number of signaling pathways, which were demonstrated to result in tumor progression.

NAP1L1 mRNA expression in human PLTs was previously described [49]; however, no function was assigned to this versatile nuclear protein in such anucleate cells, or its progenitors, MKs. Here, we demonstrate that MKs and human PLTs endogenously express differential NAP1L1 mRNA variants and protein isoforms. We identified PDC-E2, a subunit of the mitochondrial pyruvate dehydrogenase (PDH complex), as a protein interaction partner. In PLTs, septic conditions induced increased expression of NAP1L1 protein, and PDH complex activity. In contrast, exogenous overexpression of NAP1L1 in MKs resulted in reduced proplatelet formation, and decreased PDH complex activity when treated with LPS, simulating a septic environment. Our findings suggest a previously unidentified NAP1L1 interaction partner in human PLTs and MKs. Furthermore, this newly described protein–protein interaction seems to influence PDH complex activity, with subsequent functional disturbances of these specialized cells.

## 2. Results

### 2.1. CD34^+^-Derived MKs and Freshly Isolated Human PLTs Express NAP1L1

**Figure 1:**
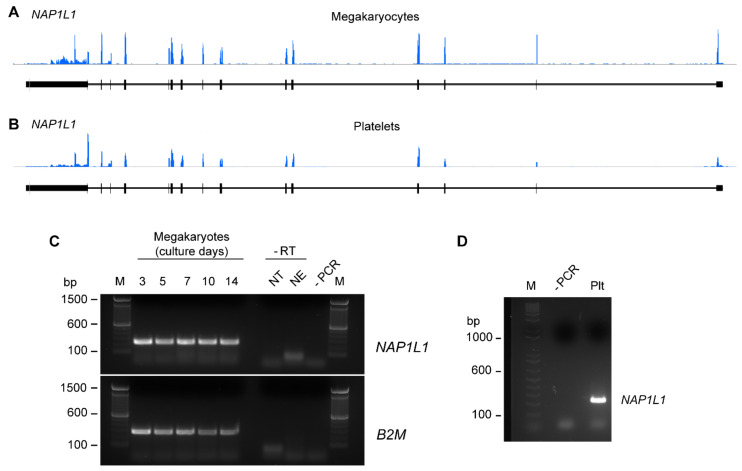
**MKs and human PLTs express mRNA for NAP1L1.** RNA-seq snapshots taken from the Integrated Genome Browser of the *NAP1L1* transcript in CD34+-derived MKs (**A**) and human PLTs (**B**) (representative for n = 3). The height on the *y*-axis represents the relative accumulated number of reads spanning a particular sequence. The average of the read depths across all genomic coordinates within a transcript correlate to abundance of RNA expression. *NAP1L1* transcript regions are represented below the plots by thick (exon) and thin (intron) lines. (**C**) MKs at culture days 3, 5, 7, 10, and 14 were examined for *NAP1L1* expression by PCR. NT represents the reverse transcription (RT) reaction using no template, NE represents the RT reaction using no enzyme. Both controls were performed to control for contaminating nuclear DNA. **Bottom**, *B2M* as a loading control. This figure is representative of 5 independent experiments. (**D**) RNA from freshly isolated PLTs at baseline was isolated and *NAP1L1* mRNA expression was evaluated by PCR. This figure is representative of 3 independent experiments. The -PCR lane shows the negative control for the PCR reaction, lanes labeled with M indicate the marker lane.

We initially sought to determine RNA expression patterns of *NAP1L1* in CD34^+^ hematopoietic progenitor cell-derived MKs and freshly isolated human PLTs. Next generation RNA-sequencing (RNA-seq) revealed that MKs and human PLTs express messenger RNA (mRNA) for *NAP1L1* (Figure 1A,B). Consistent with its expression demonstrated via RNA-seq, *NAP1L1* mRNA was also detected in MKs using PCR techniques (Figure 1C). *NAP1L1* was consistently expressed over the entire time course of MK culture and differentiation. Furthermore, *NAP1L1* was readily detectable in RNA samples isolated from human PLTs (Figure 1D). Of note, all PLT samples used for these studies were DNase treated to exclude any DNA contaminants from nucleated cells.

**Figure 2:**
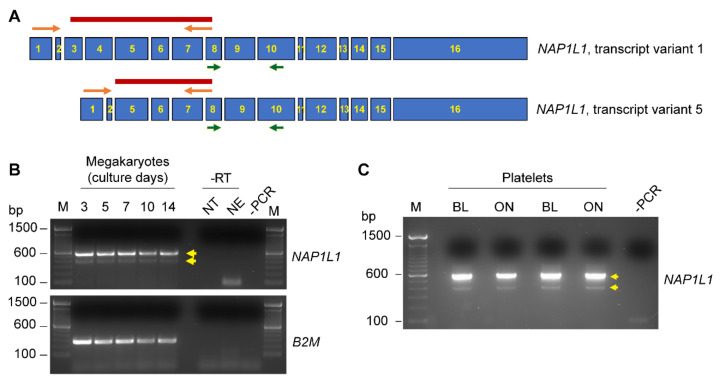
**MKs and human PLTs express alternative *NAP1L1* transcript variants.** (**A**) Schematic representation of the organization of exons in NAP1L1 transcript variant 1 (**top**), and transcript variant 5 (**bottom**) missing exons 3 and 4. Primer binding sites for transcript variant 1 detection (green, as used in Figure 1) and transcript variant 5 (orange), and the expected PCR products (dark red) are indicated. (**B**) MKs at culture days 3, 5, 7, 10, and 14 were examined for *NAP1L1* expression by PCR. NT represents the reverse transcription (RT) reaction using no template, NE represents the RT reaction using no enzyme. Both controls were performed to control for contaminating nuclear DNA. **Bottom**, *B2M* as a loading control. This figure is representative of 5 independent experiments. (**C**) RNA from freshly isolated PLTs at baseline (BL) and after culture overnight (ON) was isolated and *NAP1L1* mRNA expression was evaluated by PCR. This figure is representative of 10 independent experiments. The -PCR lane shows the negative control for the PCR reaction, lanes labeled with M indicate the marker lane. Relevant PCR products are indicated by yellow arrows.

Since multiple alternative transcripts are described to exist for *NAP1L1*, we next performed PCR experiments using a primer pair spanning from exon 1 to exon 7, enabling us to detect a potential alternative *NAP1L1* transcript variant 5, which is devoid of exons 3 and 4 (Figure 2A). MKs as well as PLT samples demonstrated the presence of PCR products indicative of *NAP1L1* transcript variant 1 (Figure 2B,C), PCR product band indicated at ~600 bp) and transcript variant 5 (Figure 2B,C, PCR product band indicated at ~400 bp). These findings were verified by Sanger sequencing for excised PCR products, demonstrating full length transcript as well as a transcript devoid of exon 3 and 4 (data not shown). In addition, we were able to detect *NAP1L1* transcript variants at all stages of MK differentiation (Figure 2B). Furthermore, allowing the incubation of PLTs overnight (Figure 2C), or the placement of PLTs on immobilized fibrinogen and stimulating the cells using thrombin (Supplementary Figure S1) did not alter the expression pattern of alternative transcripts; therefore, no loading control was included.

**Figure 3:**
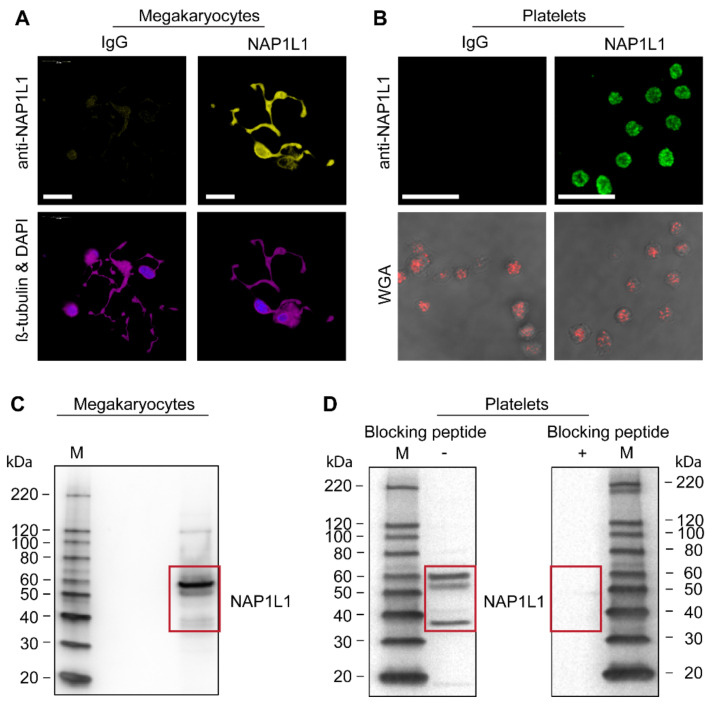
**MKs and human PLTs express NAP1L1 protein.** (**A**) CD34^+^-derived MKs were placed on immobilized fibrinogen to induce proplatelet formation, fixed, and then incubated with an antibody against NAP1L1 (yellow, top right panel) or an IgG control, followed by co-staining for b-tubulin (magenta) and nuclei (40,6-diamidino-2-phenylindole, DAPI, blue), as shown in the bottom panels. Scale bars = 20 µm. This figure is representative of n = 7 independent experiments. (**B**) NAP1L1 protein expression (green) in human PLTs. PLTs incubated with an IgG control antibody are shown (left, IgG). The bottom panels show the corresponding transmission images overlayed with the co-staining with WGA (red). Scale bars = 10 µm. This figure is representative of n = 6 independent experiments. (**C**) Immunoblot analysis of MK lysates (day 13 of culture) using the anti-NAP1L1 antibody. The cells were lysed, and proteins separated by SDS-PAGE electrophoresis. NAP1L1 isoforms are highlighted by a red box. (**D**) Immunoblot analysis of PLT lysates using the anti-NAP1L1 antibody. The cells were lysed, and proteins separated by SDS-PAGE electrophoresis; however, the samples were split and processed in parallel in the absence (**left**) or presence (**right**) of an NAP1L1-quenching peptide. The presence of the blocking peptide prevents the detection of NAP1L1 with the anti-NAP1L1 antibody, demonstrating specificity for the three main NAP1L1 protein isoforms. Lanes labeled with M indicate the marker lane. (**C**,**D**), representative of 3 and 8 independent experiments, respectively.

Consistent with our findings by PCR and Sanger sequencing (Figure 1 and Figure 2, and data not shown), we found that NAP1L1 protein is endogenously and robustly expressed in situ in the cytoplasm and proplatelet extensions of MKs (Figure 3A). It is interesting to note that NAP1L1 protein localization in MKs is less pronounced in cell nuclei; however, it demonstrates some peri-nuclear enhancement. Furthermore, cells undergoing anaphase showed reduced NAP1L1 association with condensed chromosomes (Supplementary Figure S2). While nucleosome assembly proteins were initially identified as being histone chaperones and chromatin-assembly factors, additional functions were assigned including tissue-specific transcription regulation, apoptosis, histone shuttling, cell-cycle regulation, and additional chaperone functions beyond histone binding [41]. Our microscopic findings in MKs could potentially point towards such extra nuclear functions.

In unstimulated human PLTs, NAP1L1 protein concentrated and accumulated peri-granular (when compared with WGA co-staining) in a fine vesicular pattern (Figure 3B).

Immunoblotting also confirmed our microscopic findings of NAP1L1 protein being present in MKs (Figure 3C). Surprisingly, Western blotting revealed three major protein bands at 60, 53 and 37 kDa, while the predicted size for NAP1L1 protein (expected isoform 1) is 43–53 kDa. Therefore, we sought to examine the specificity of the used anti-NAP1L1 antibody. A peptide competition assay (quench experiment) using the immunogenic peptide (Figure 3D, right) showed that all three detected protein bands specifically represent NAP1L1 isoforms or potentially post-translational modified NAP1L1 in human PLT samples (Figure 3D, left).

### 2.2. NAP1L1 Directly Interacts with the Dihydrolipopolylysine-Residue Acetyltransferase Component of Pyruvate Dehydrogenase Complex (PDC-E2, ODP2)

**Figure 4:**
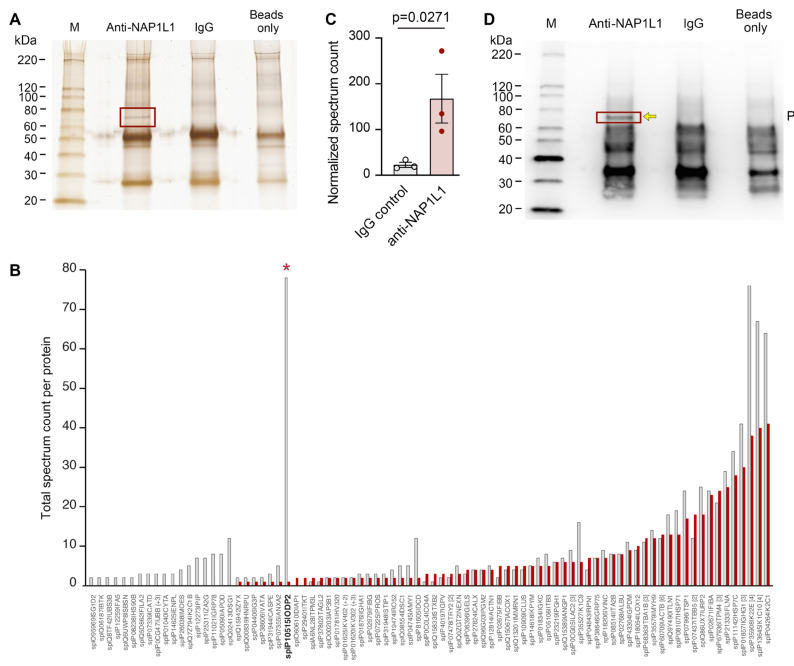
**NAP1L1 directly interacts with PDC-E2.** (**A**) Sodium dodecyl sulfate (SDS) gel were stained for total protein using standard MS-compatible silver staining. The left lane shows proteins isolated after co-immunoprecipitation (co-IP) using an anti-NAP1L1 antibody. The middle lane shows proteins isolated after co-IP with non-immunogenic, control IgG. The right lane shows proteins isolated after the use of protein-G beads only. Red box marks selected protein band subsequently used in MS analysis. This figure is representative of n = 5 independent experiments. (**B**) Bar graph showing the total spectrum count per protein. Black bars show counts for MS performed after control IgG experiment; non-filled bars demonstrated spectrum counts for proteins identified after co-IP using anti-NAP1L1. Total spectrum counts for PDC-E2 are indicated by red asterisk. (**C**) Bar graph showing the normalized spectrum count (normalized to all total spectrum counts) for PDC-E2. Black bar represents counts for MS performed after control IgG experiment; non-filled bar show spectrum counts for PDC-E2 identified after co-IP using anti-NAP1L1. This figure is representative of n = 3 independent experiments. Analyzed using *t*-test, *p* = 0.0271. (**D**) A corresponding sodium dodecyl sulfate (SDS) gel was analyzed using immunoblotting technique. Co-IP proteins were probed using an anti-PDC-E2 antibody. The left lane shows proteins isolated after co-immunoprecipitation (co-IP) using an anti-NAP1L1 antibody. The middle lane shows proteins isolated after co-IP with non-immunogenic, control IgG. The right lane shows proteins isolated after the use of protein-G beads only. Red box and yellow arrow mark PDC-E2 protein detected as being co-IP’ed using the anti-NAP1L1 antibody, indicating protein–protein interaction. This figure is representative of n = 3 independent experiments.

Because of its multitude of functions described in the literature, and its widely unknown roles in MK and PLT cell biology, we next wanted to identify NAP1L1 protein binding partners in human PLTs. To identify such interaction partners, we used carefully controlled co-IP experiments followed by mass spectroscopy (MS) analyses of a selected band. We identified a highly specific protein, which was only present in the specific anti-NAP1L1 pull-down (Figure 4A, second lane from left); however, was absent in the isotype-specific IgG control (Figure 4A, third lane from left). Furthermore, we excluded non-specific binding to the protein G magnetic beads by including a control condition using those beads only (Figure 4A, right lane).

Besides contaminating human cytokeratin, which was most likely introduced during protein processing, we found the highest total spectrum count being present for ODP2_HUMAN (Figure 4B), the human mitochondrial dihydrolipoyllysine-residue acetyltransferase component (PDC-E2). The highly specific nature of this identified NAP1L1 binding partner was further underscored by its significantly increased normalized spectrum count for peptide sequences, when compared to samples co-IPed using IgG (Figure 4C). Consistent with its expression pattern at the peptide level, immunoblotting demonstrated robust and specifically detectable NAP1L1—PDC-E2 protein interaction in human PLTs (Figure 4D) using Western blot approaches.

PDC-E2—human mitochondrial dihydrolipoyllysine-residue acetyltransferase is the E2 component of the multi-enzyme pyruvate dehydrogenase complex (PDC). In nucleated cells, DLAT, the gene encoding PDC-E2, is part of the nuclear DNA, and upon translation, the protein needs to be transported to the inner mitochondria membrane. Here, the enzyme transfers acetyl groups to coenzyme A. In addition, PDC can also be present in the nucleus catalyzing histone acetylation [50,51]. In turn, PDC-E2 is also the key antigen in primary biliary cholangitis, a chronic liver disease with autoimmune component targeting intrahepatic bile duct lining cells [52,53].

### 2.3. NAP1L1 and PDC-E2 Show Dynamic Expression Changes When PLTs Are Exposed to Septic Conditions

NAP1L1 was previously assigned chaperone and transport functions, involving histone complexes [54,55], diacylglycerol kinase ζ [56], mtHsp70 [57]. Here, we add an additional interaction partner, PDC-E2, to the evolving functional repertoire of NAP1L1. With PDC-E2 being an integral part of the mitochondrial pyruvate dehydrogenase complex, we started exploring functional consequences of the NAP1L1–PDC-E2 interaction. Several studies demonstrated that the mitochondrial pyruvate dehydrogenase complex gets dysregulated due to septic host environment [58,59]. Although anucleate, emerging data demonstrates that the human PLT transcriptome is not static. Rather, the PLT transcriptome is dynamic and markedly altered during systemic inflammatory diseases [6,7,22,26]. Therefore, we sought to examine NAP1L1 and PDC-E2 protein expression levels in human PLTs isolated from septic patients.

**Figure 5:**
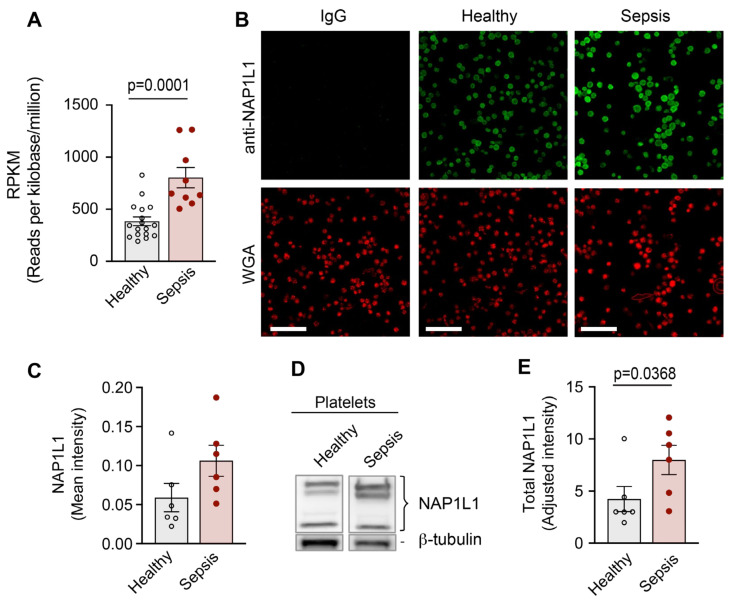
**NAP1L1 shows dynamic protein expression changes when PLTs are exposed to septic conditions.** PLTs were isolated from healthy individuals or septic patients within 48 h of ICU admission. (**A**) Total RNA was isolated and next generation RNA-sequencing (RNA-seq) was performed (n = 9, and 17, for septic and healthy, respectively). Data show the RPKM expression for NAP1L1 for healthy (grey), and septic (non-filled) samples. Analyzed using *t*-test, *p* = 0.0001. (**B**) PLTs were fixed in suspension immediately after isolation. Immunofluorescence staining with an anti-NAP1L1 (green) antibody demonstrates robust expression of NAP1L1 protein in PLTs from septic patients and healthy PLTs (scale bars: 10 µm). Cells were co-stained using WGA (red). This figure is representative of n = 6 independent experiments. (**C**) Micrographs were analyzed using CellProfiler. The bar graph displays the mean intensity over all stained cells for NAP1L1, normalized to IgG. Analyzed using *t*-test, *p* = 0.0505. (**D**) PLTs from healthy individuals and septic patients were isolated. NAP1L1 protein expression levels were detected by Western blot. Positions of NAP1L1 isoforms are marked. Corresponding tubulin is shown at the bottom and was used for normalization of data presented in bar graph shown in (**E**) below. This figure is representative of n = 6 independent experiments. Analyzed using Mann–Whitney test, *p* = 0.0368.

To determine whether transcripts coding for NAP1L1 were altered in PLTs during sepsis, we used RNA-seq on highly purified PLTs from septic patients and matched healthy donors. This study was performed as a sub-analysis of a dataset previously published by our group [8]. Figure 5A demonstrates the significant increase in mRNA coding for *NAP1L1* in PLTs isolated from septic patients.

Having detected significantly increased *NAP1L1* transcript levels in PLTs from septic patients, we hypothesized that NAP1L1 protein expression would similarly be altered during sepsis. We initially used immunofluorescence staining (ICC) to examine NAP1L1 protein expression and localization in PLTs from septic patients and matched healthy control donors. NAP1L1 staining was localized peri-granular (when compared with WGA co-staining) in a fine vesicular pattern (Figure 5B). In septic PLTs, using automated image analysis, we detected an increased mean fluorescent intensity for NAP1L1 (Figure 5C), which translated into a significant fold-increase of staining intensity (data not shown).

To further test the above hypothesis, we used Western blot analysis to examine total PLT NAP1L1 protein expression. Figure 5D,E demonstrate significantly increased NAP1L1 protein expression in PLTs from septic patients.

**Figure 6:**
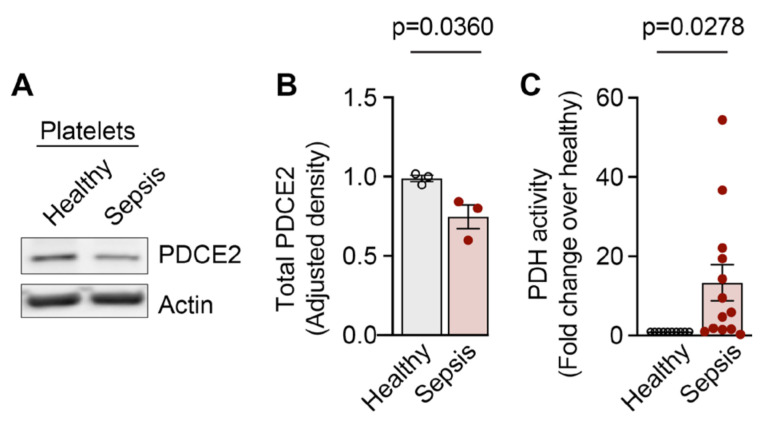
**PDC-E2 protein levels and pyruvate dehydrogenase complex activity is increased in PLTs during sepsis.** PLTs from healthy individuals and septic patients were isolated. (**A**) PDC-E2 protein expression levels were detected by Western blot. Position of PDC-E2 is marked. Corresponding actin (ACTB) is shown at the bottom and was used for the normalization of data presented in the bar graph shown in (**B**) below. This figure is representative of n = 3 independent experiments. Analyzed using *t*-test, *p* = 0.0360. (**C**) Pyruvate dehydrogenase complex activity assay was performed on PLTs isolated from septic (n = 13) and healthy (n = 11) individuals. Bar graph depicts mean optical signal ± SEM. Analyzed using *t*-test, *p* = 0.0278.

In contrast to the increased NAP1L1 expression, Western blot analysis for PDC-E2 showed significantly decreased total protein expression in PLTs isolated from septic patients when compared to healthy controls (Figure 6A,B).

### 2.4. Pyruvate Dehydrogenase Complex Activity Is Increased in PLTs during Sepsis

The immunometabolism during sepsis is characterized by a switch from a high-energy infection resistance mode to a low-energy conservation tolerance mode within 4–8 h after sepsis dissemination [58]. The pyruvate dehydrogenase complex acts as an energy homeostat, and its deactivation creates an energy supply chain shortage during sepsis. Here, pyruvate dehydrogenase (PDH) complex activity was significantly increased in PLTs isolated from septic patients when compared to healthy donor PLTs (Figure 6C), which could indicate an early stage in the septic continuum.

### 2.5. NAP1L1 Overexpression in MKs Induced Reduced Proplatelet Formation (PPF)

To further establish potential functional consequences of the newly described NAP1L1–PDC-E2 protein interaction, we utilized an established CD34^+^-derived human MK cell culture model [23,24,31,60].

**Figure 7:**
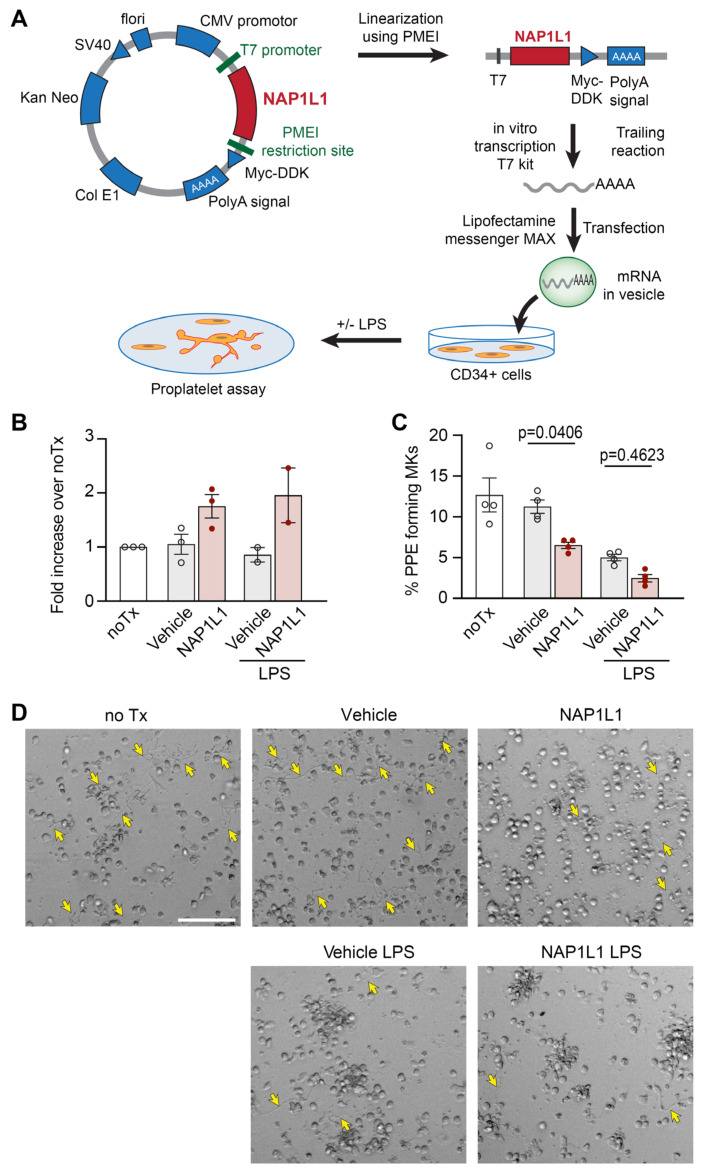
**NAP1L1 overexpression in MKs results in decreased (PPF).** (**A**) Schematic of protocol for NAP1L1 overexpression in MKs. Functional elements of the NAP1L1 expression plasmid are demonstrated. (**B**) Immunoblot analysis of MK lysates (day 13 of culture) using the anti-NAP1L1 antibody. Cells were lysed and proteins separated by SDS-PAGE electrophoresis. Samples undergoing transfection with the in vitro transcribed NAP1L1 mRNA are labeled with NAP1L1; LPS-treatment groups are indicated. The bar graph shows a fold increase over non-treated cells (NoTx). This figure is representative of n = 2/3 independent experiments, as indicated by single data points. Analyzed using one way ANOVA, followed by Tukey’s multiple comparison, *p* = 0.0310. (**C**) Plot of percentages of day 13 MKs with PPs after being not treated (noTx), treated with LPS, or undergoing NAP1L1 transfection (NAP1L1). Asterisks indicate a significant decrease in PPF when compared to noTx and vehicle. This figure is representative of n = 4 independent experiments. Analyzed using one way ANOVA, followed by Tukey’s multiple comparison, noTx vs. vehicle, *p* = 0.8687, noTx vs. NAP1L1, *p* = 0.0064, noTx vs. vehicle LPS, *p* = 0.0009, noTx vs. NAP1L1 LPS, *p* =< 0.0001, vehicle vs. NAP1L1, *p* = 0.0406, vehicle vs. vehicle LPS, *p* = 0.0058, vehicle vs. NAP1L1 LPS, *p* = 0.0002, NAP1L1 vs. vehicle LPS, *p* = 0.8467, NAP1L1 vs. NAP1L1 LPS, *p* = 0.0992, vehicle LPS vs. NAP1L1 LPS, *p* = 0.4623. (**D**) Representative brightfield images of MKs with PPs (yellow arrows) after *NAP1L1* overexpression, with or without LPS treatment. Scale bar: 50 µm.

MKs are PLT precursor cells, contain many of the same proteins and pathways as PLTs, and are often used as a relevant model system to study PLT responses [8,60,61]. While PLTs are only extremely difficult or not at all accessible to modifications of their transcriptome, MKs have been genetically modified using several different techniques [62,63]. Here, we used “liposome-based transfection” utilizing the transfer of in vitro transcribed, capped, and poly(A) tailed *NAP1L1* mRNA into MKs (Figure 7A), to induce transient overexpression of NAP1L1. Figure 7B demonstrates the successful overexpression of NAP1L1 protein in MKs. In contrast to PLTs isolated from septic patients, the exposure of human MKs to an agonist commonly generated during sepsis (lipopolysaccharide, LPS) did result in a slight decrease of the endogenous NAP1L1 protein level, independent of the transfection. In addition, treatment of transfected cells with LPS did also not lead to NAP1L1 protein levels above the non-LPS but transfected condition. We next analyzed proplatelet formation (PPF), an energy intensive hallmark functional determinate of MKs in NAP1L1 transfected cell cultures. We were able to show that NAP1L1 protein overexpression in non-treated cells, and even more pronounced in with LPS-treated cells, significantly decreased PPF (Figure 7C,D). Additional co-IP experiments using MKs demonstrated increased NAP1L1–PDC-E2 interaction when cells were exposed to LPS (Supplementary Figure S3A). Furthermore, consistent with the PPF data indicating decreased functional capacity of MKs under septic conditions, LPS treatment decreased the pyruvate dehydrogenase complex activity in our culture model (Supplementary Figure S3B).

## 3. Discussion

The MK-PLT axis is increasingly recognized as being more intricate and sophisticated in cellular responsibilities and abilities than historically expected. MKs are not only producing PLTs in the bone marrow through thrombopoiesis but are also intimately involved in setting up the PLT immune potential. MKs invest PLTs with the molecular composition to take center stage as responders and immune sentinels in the initiation and propagation of coordinated host immune responses [64]. Furthermore, while PLTs are anucleate, it has been demonstrated that they contain a complex transcriptome [20,49] and regulatory pathways, including cytoplasmic splicing, controlling important biologic tasks [22,23,24,26,27,28,60,65,66,67]. In concert with these signal-dependent pathways, it was demonstrated that PLTs possess nuclear proteins. In a comprehensive PLT proteome analysis, 13% of all identified proteins were mapped to a nuclear location [68]. PLTs utilize such proteins in established functions, as well as assigning alternative roles. Amongst other nuclear proteins, MKs distribute a functional spliceosome to PLTs [23,24]. In addition, nuclear receptors, including the receptors for sex steroids, glucocorticoids, peroxisome proliferator-activated receptors (PPAR)s, and retinoid X receptors (RXR)s were detected in PLTs, reported to bind their respective ligands, which induce PLT aggregation and activation (reviewed in [69]). Unexpectedly, RAR was also demonstrated to be utilized by PLTs as a translational regulator [28]. RAR is additionally employed in non-genomic activities by controlling actin cytoskeletal events [31]. Nevertheless, there is paucity of data exploring and finally comparing the functional capacities of nuclear proteins in PLTs with their counterparts residing in MKs.

Here, we demonstrate for the first time, to the best of our knowledge, that *NAP1L1* mRNA is expressed in PLTs demonstrating potential transcript variants previously not detected in PLTs (Figure 1 and Figure 2, and Supplementary Figure S1). Furthermore, we identify a protein interaction partner, PDC-E2, hitherto not characterized as being part of the NAP1L1 protein network (Figure 4). Using septic PLTs, we were able to also demonstrate a dynamic protein expression pattern of NAP1L1 and its interaction partner PDC-E2, potentially resulting in altered pyruvate dehydrogenase complex (PDH) activity (Figure 5 and Figure 6). In addition, using human MK cells, we were able to show that overexpression of NAP1L1 results in diminished proplatelet formation, and decreased PDC-E2-dependent PDH activity (Figure 7 and Supplementary Figure S3).

Our analyses of MKs and PLTs are also the first to examine *NAP1L1* transcript levels and protein expression pattern. While *NAP1L1* mRNA was previously detected in human PLTs [49,70], no in-depth analysis of potential transcript variants, changes in transcript levels, or specific protein expression or localization analysis was performed. Consistent with previous RNA sequencing results, we observed expression of *NAP1L1* mRNA using RNA sequencing techniques as well as confirmatory PCR approaches for PLT as well as MK samples, harvested throughout the MK culture period (Figure 1). Next, we designed a PCR primer pair spanning from exon 1 to exon 7. This enabled us to detect alternative transcript processing in MKs and PLTs resulting in *NAP1L1* transcript variant 5 (NM_001330232.2), which is devoid of exons 3 and 4 (Figure 2). This suggests that alternative splicing of *NAP1L1* occurs in MKs, and that PLTs get invested with such alternative transcripts by MKs. It is interesting to note that the transcript variant ratios did not change due to activation of PLTs using immobilized fibrinogen and thrombin (Supplementary Figure S1), indicating that the alternative splicing process does not occur in the PLT cell bodies. *NAP1L1* transcript variant 5 is coding for the putative NAP1L1 isoform 3 (NP_001317161.1), which misses the N-terminal domain I. Domain I is mainly responsible for dimerization of NAP1L1 and mediating histone interaction [54,55,71]. The presence of transcript variant 5, coding for a protein with a deleted functional domain, could be indicative of alternative protein functions of NAP1L1, besides the nucleosome assembly task in MKs and human PLTs. Taken together, these initial findings focused on *NAP1L1* transcripts demonstrated the presence of at least two *NAP1L1* transcript variants (1 and 5) and raised the question regarding whether NAP1L1 protein isoforms are located in MK and PLT cell bodies.

Using initial microscopy studies, we were able to demonstrate NAP1L1 protein in MKs, their proplatelet extensions (Figure 3A), as well as in PLT cell bodies in a peri-granular pattern. While not performing detailed co-localization studies, the images from PLT studies support the finding that NAP1L1 protein does most likely not reside within the PLT granular compartment (Figure 3B). Interestingly, NAP1L1 protein signals demonstrate minimal co-localization with nuclear/DNA co-staining signals when examining dividing MK-progenitors (Supplementary Figure S2). Despite the known NAP-family members functions in DNA replication and cell proliferation [72], and the previously described changing intracellular localization [41], this could furthermore point towards an alternative cellular function assigned to NAP1L1 in the MK-PLT axis.

To confirm our microscopy data, we next examine NAP1L1 protein expression using Western blot analysis (Figure 3C,D). Our studies specifically demonstrate, for the first time, the presence of at least three different NAP1L1 isoforms in MKs and PLTs. This finding is consistent with the initial description of NAP1L1 and subsequent publications, which showed Western blot results indicating the existence of multiple NAP1L1 protein isoforms [40,43,73]. To exclude cross-reactivity of the detection antibody, which could lead to false positive results, we performed peptide competition assays clearly demonstrating the specificity of our findings. The difference between observed and predicted molecular weight for NAP1L1 proteins, shown in our study, can be explained by potential post-translational modifications. NAP1L1, as part of the human NAP1L-family members, is known to contain C-terminal glutamylation sites, creating a reversible acidic C-terminal moiety, with the potential of switching between different protein functions [55,74,75], but also resulting in changed migration during SDS-PAGE. This potential mechanism of post-translational protein and also functional modification was highlighted in a recent publication [76]. Ye and colleagues demonstrated that MKs contain the enzymatic machinery necessary for performing and reversing polyglutamylations, namely tubulin tyrosine ligase-like family (TTLL) members TTLL4 and TTLL6, and cytosolic carboxypeptidase (CCP) 6. Therefore, differential protein isoform as well as specific post-translational modifications could be displayed by the detected NAP1L1 signals.

To explore additional NAP1L1 functions, besides the traditionally assigned roles, we wanted to further explore if new protein partners interacting with NAP1L1 could be identified in MKs and PLTs. Using co-IP techniques, we were able to reproduce a highly specific protein band, which could be identified and verified as PDC-E2, the protein product of *DLAT* (Figure 4). PDC-E2 is part of the mitochondrial multi-enzyme pyruvate dehydrogenase (PDH) complex (PDC), which converts pyruvate to acetyl-CoA. Recent studies indicated that PDC is also present in the nucleus and processes/catalyzes histone acetylation [51,77], and its subunit PDC-E2 can act as a nuclear transcriptional regulator [78]. PDC-E2, also called dihydrolipoamide acetyltransferase, is the acceptor molecule for acetyl groups formed by the oxidative decarboxylation of pyruvate and transfers them to coenzyme A. Of note, in addition to its essential role in cellular metabolism, PDC-E2 is the key autoantigen in primary biliary cirrhosis (PBC) [53]. Like many mitochondrial proteins, PDC-E2 is encoded by the nuclear genome and translated in the cytoplasm with an amino-terminal targeting sequence [79,80]. A direct interaction of NAP-family members with mitochondrial proteins is not unprecedented. Using a yeast two-hybrid system, Stelzl and colleagues created extensive protein–protein interaction maps, demonstrating that NAP1L1 interacted with dihydrolipoamide succinyltransferase (E2), a component of the mitochondrial 2-oxoglutarate dehydrogenase complex [81]. Furthermore, Okada et al. [56] demonstrated that NAP1L1 interacted with diacylglycerol kinase ζ (DGKζ), which prohibited its nuclear import through blocking import carrier protein interaction. Therefore, in MKs, one might speculate if the NAP1L1–PDC-E2 interaction could serve a similar import–export control pathway.

Because of its clinical relevance, we decided to study NAP1L1 expression and potential function in a common, morbid, and often fatal systemic inflammatory disease—sepsis. We detected an increase in *NAP1L1* transcript in human PLTs, a finding which was paralleled by the protein expression data shown via immune detection of NAP1L1 (Figure 5). These findings pointed towards a dynamic regulation of NAP1L1 in septic PLTs, a clinical situation NAP1L1 was never studied under. Next, we examined PDC-E2 protein expression pattern in septic PLTs (Figure 6). PDC-E2 protein expression levels decreased in septic PLTs. However, PDH complex activity increased 13-fold over the metabolic activity measured in PLTs isolated from healthy individuals. This finding could be met with surprise since published studies indicate that the PDH complex is inhibited during sepsis, resulting in a switch from mitochondrial aerobic oxidation to cytoplasmic anaerobic glycolysis, a process known to contribute to the formation of lactic acid and intracellular acidosis, which can lead to intracellular Ca2^+^ overload, mitochondrial membrane damage [58,82,83]. Nevertheless, contrasting the general PDC-dependent metabolic reprogramming response in sepsis [58,84,85], several studies demonstrated that mitochondrial oxygen consumption in PLTs is increased in septic patients, and closely related to their survival rate [86,87,88]. Therefore, based on our observations, we speculate that due to its chaperone and potential shuttling function, NAP1L1 protein in septic PLTs could help stabilize the conformation of PDC-E2 molecules, and also mediate the known one-way transport [89] into the mitochondrial compartment.

To confirm these results, the next step would be to genetically modify the target cells to demonstrate functional coherence. However, it is difficult to produce transfected and genetically modified uniform, high-quality human PLTs. Therefore, to further decipher the functional and mechanistic impact of NAP1L1–PDC-E2 interaction on the MK-PLT axis, we utilized a well-established PLT-surrogate MK culture model, as previously used by our laboratory [7,23,24,31]. To mimic increased NAP1L1 protein levels in septic PLTs, we used “liposome-based transfection” techniques (Figure 7A). Using this approach, NAP1L1 could be robustly overexpressed in the human MK culture system (Figure 7B). Transfected or vehicle-treated MKs were subsequently incubated with LPS, one of the major determinants in Gram-negative sepsis [90]. It is important to note that LPS uses a well-established signaling pathway through TLR4, which is expressed by CD34^+^-derived MKs [36]. In contrast to our septic PLT experiments, we observed that LPS treatment alone resulted in slightly decreased NAP1L1 expression levels. Performing a proplatelet formation (PPF) assay [17], a standard read-out for MK function, we detected decreased PPF when NAP1L1 was overexpressed in MKs. In addition, it appeared that NAP1L1 protein overexpression and simulated septic conditions using LPS treatment, synergistically reduced PPF (Figure 7C,D). During their final stages, MKs produce PLTs by extending long cytoplasmic protrusions, designated proplatelets [9], into sinusoidal blood vessels, a process coined PPF. While two independent, but interrelated cytoskeletal systems, actin filaments and tubulin subunits, provide the force leading to the shape changes associated with MK maturation and proplatelet formation [10], it was elegantly shown that dynein-dependent microtubule sliding acts as the driving force for proplatelet elongation and PLT biogenesis [11]. The rolling up of microtubules as they drive apart and extend the proplatelet is likely to be involved in forming the coils found in the proplatelet tips and the mature PLTs, called marginal band. The production of force with its synchronized cycles of microtubule binding and release, is energy consuming, using the hydrolysis of ATP [12]. Therefore, we sought to examine NAP1L1–PDC-E2 interaction and PDH complex activity in MKs under simulated septic conditions, which might explain the decreased PPF. LPS-treated MKs demonstrated increased interactions between NAP1L1 and PDC-E2, and a significantly decreased PDH complex activity (Supplementary Figure S3). These findings contrast our experimental results from septic PLTs. Nevertheless, opposite NAP1L1–PDC-E2 interaction levels, and PDH complex activity could potentially be explained by PLTs being anucleate, and therefore potentially using NAP1L1 protein in a different context than nucleated MKs. In PLTs, it seems more likely that NAP1L1 function could be defined and garnered towards chaperoning PDC-E2. Contrastingly, in MKs, NAP1L1 could help in the compartmentalization of PDC-E2, and therefore prohibiting the protein from entering its functional complex PDC, as it was shown for DGKζ [56]. In sepsis, thrombocytopenia is closely related with clinical outcome. In this context, our MK data also complements previous reports demonstrating that one potential mechanism for low PLT counts in severely ill patients with advanced thrombocytopenia could be diminished thrombopoiesis [91,92]. Therefore, it is interesting to speculate about potential upstream signaling mechanisms triggering, and potentially enabling the NAP1L1–PDC-E2 protein interaction. In 1999, Cataldo and colleagues reported that Mpl (thrombopoietin, TPO), ligand of the Mpl-c receptor, regulates NAP1 family member expression in a rat bone marrow model [93]. The authors were able to demonstrate that these NAP-related effects were limited to Mpl and could not be reproduced by other bone marrow growth factors (i.e., granulocate colony stimulating factor), which led to the conclusion of a cytokine specific effect. These results can be interpreted as a TPO-specific signaling pathway integrating sensing of PLT numbers, stimulation of progenitor cells by Mpl, and subsequent modulation of protein interactions. The involvement of the Mpl (TPO) signaling pathway in the newly discovered NAP1L1–PDC-E2 protein interaction will therefore be a focus of future studies.

The presented study was data driven and performed under best research practice. Nevertheless, there are some limitations to this study. Since the generation of the presented data, CRISPR-Cas9 technique has been developed for rapid genetic manipulation of MKs [62]. For future studies we would propose to use such an approach for further dissecting the mechanistic function of PDC-E2 in MKs in greater detail. We appreciate the descriptive character of our study; however, this is the first report of NAP1L1 interacting specifically with a previously unrecognized partner protein. Therefore, we recognize that our findings should be extended in future studies, potentially complemented by in vivo work using relevant mouse models.

In conclusion, our data demonstrate NAP1L1 expression in human PLTs and MKs. This is the first time that expression of different *NAP1L1* transcript variants, and protein isoform was confirmed in such cells. Our findings demonstrate, for the first time, that NAP1L1 interacts with PDC-E2, a subunit of the PDH complex. The PDH complex acts as an energy homeostat bridging glycolysis to energy production, and its function gets modulated during sepsis, carbohydrate metabolism away from mitochondrial oxidation. Our data shed new light on such processes taking place in PLTs and MKs during sepsis. At the moment, however, it remains undetermined how NAP1L1 functions are coordinated when comparing nucleated versus anucleate state. Nevertheless, our results support the conclusion of differential functions of NAP1L1 in MKs and PLTs during systemic inflammation and open a new avenue of sepsis research focused on the MK-PLT axis.

## 4. Materials and Methods

### 4.1. Reagents and Antibodies

The following reagents and antibodies were used for microscopy, Western blot, and co-IP studies as described below: Paraformaldehyde (PFA 4% [2% final]), Alexa Fluor® 555 conjugate of wheat germ agglutinin (WGA, 1:1000; Thermo Fisher Scientific, Waltham, MA, USA, W32464), Alexa Fluor® 647 conjugate of phalloidin (Thermo Fisher Scientific, A22287), 4′,6-Diamidino-2-Phenylindole, Dihydrochloride (DAPI, Thermo Fisher Scientific, D1306), mouse anti-NAP1L1 antibody (abcam, Cambridge, MA, USA, ab58677), rabbit anti-NAP1L1 (abcam, ab33076), rabbit anti-Pyruvate dehydrogenase complex component E2 (PDC-E2, ODP2; abcam, ab 66511), rabbit anti-beta tubulin (abcam, ab232361), mouse anti-tubulin (MilliporeSigma, Billerica, MA, USA, T-5293), mouse anti-ACTB/actin HRP-conjugated (abcam, ab20272), and NAP1L1 blocking peptide (abcam, ab22418). Secondary HRP-conjugated antibodies, and secondary antibodies conjugated to Alexa Fluor® 488 and 546 were purchased from Thermo Fisher Scientific (G-21040, G-21234, S11223, A-11001, A-11008, A-21422, A-21428).

### 4.2. Septic Patients

Patients were selected for meeting consensus criteria for sepsis [94], and enrolled within 48 h of ICU admission (n = 9, and 17, for septic and healthy, respectively).

### 4.3. PLT Isolation and Culture

PLTs used for the described studies were freshly isolated. PLTs were leukocyte-reduced and isolated, as previously described to yield a highly purified population of cells with <1 leukocyte per 10^7^ PLTs [23,24,66]. In addition, PLT activation during centrifugation steps was mitigated by the addition of 100 nM prostaglandin E1 (PGE-1) before each centrifugation step. Depleted PLTs were resuspended at 1 × 10^8^/mL in a serum-free M199 medium (Thermo Fisher Scientific, 11150067), placed in round-bottom polypropylene tubes, and incubated, where indicated, in a 37 °C humidified incubator at 5% CO_2_ for different time points. In select studies, PLTs were placed on immobilized fibrinogen and stimulated with thrombin (factor IIa), as previously described [24].

### 4.4. CD34^+^-Derived MKs

CD34^+^ hematopoietic progenitor cells were isolated from human umbilical cord blood and differentiated into proplatelet-producing MKs, as previously described [23,24,31].

### 4.5. RNA Isolation

MKs or PLTs were lysed in TRIzol (Thermo Fisher Scientific, 15596026), and RNA isolation was performed, as previously described [23,24]. All RNA samples were DNase treated using the TURBO DNA-free Kit (Thermo Fisher Scientific, AM 1907) per manufacturer’s protocol.

### 4.6. Next Generation RNA-Sequencing

For next-generation RNA-sequencing (RNA-seq), 1 × 10^9^ isolated PLTs were carefully lysed in TRIzol and DNase treated, as described above. Total RNA was isolated, as previously described [22,25,31,49,95]. An Agilent bio-analyzer 2100 (Agilent, Santa Clara, CA, USA) was used to quantify the amount and quality of the total RNA. RNA Integrity Number (RIN) scores were similar between all samples. RNA-seq libraries were prepared with TruSeq V2 with oligo-dT selection (Illumina, San Diego, CA, USA, RS-122-2001). Reads were aligned (Novoalign) to the reference genome at the time of these studies (GRCh37/hg19) and a pseudo-transcriptome containing splice junctions. The Deseq2 analysis package was used to assign reads to composite transcripts (one per gene) and quantitate reads per kilobase of transcript per million mapped reads/RPKMs.

### 4.7. PCR Studies

To determine *NAP1L1* mRNA expression pattern in human PLTs and CD34^+^-derived MKs, primers spanning exon 8–10 (248 bp product) of *NAP1L1* (NAP1L1_1012_forward 5′-AGT GAA GTT CTC AGA TGC TGG-3′ and NAP1L1_1259_reverse 5′-CAC GTC CCT TGT GTT TCT GC-3′) and primers spanning exon 1/2–7/8 (588 bp and 399 bp products) of *NAP1L1* (NAP1L1_460_forward 5′-AGT CGC CGA TAT TTG GAG TTC T-3′ and NAP1L1_1047_reverse 5′-TCC TGA ACC ATA TCA CTG AGC A-3′) were used (in-house synthesis) and PCR was performed. Loading control primers detecting *B2M* mRNA were used on paralleling samples (B2M_34_forward 5′-AGC TGA CAG CAT TCG GGC-3′ and B2M_333_reverse GTG GGG GTG AAT TCA GTG TAG T-3′). Annealing temperatures were selected as follows: primers spanning exon 8–10 of *NAP1L1*, 60 °C; primers spanning exon 1/2–7/8 of *NAP1L1*, 58 °C; primers detecting *B2M,* 57 °C.

### 4.8. Immunocytochemistry

On day 13 of the CD34^+^- differentiation process, MKs were placed on fibrinogen-coated chamber slides to induce proplatelet formation, fixed (PFA), permeabilized with 0.1% Triton X-100 (Thermo Fisher Scientific, AC327372500) for 5 min at room temperature (RT), and subsequently incubated with IgG, or an antibody against NAP1L1, and anti-b-tubulin antibody for co-staining purposes, overnight at 4 °C. The next day, cells were incubated with secondary antibodies conjugated to Alexa Fluor^®^ 488 or 546, followed by co-staining using DAPI for nuclear localization. In select studies (see Supplementary Figure S2), MKs were incubated with IgG or an antibody against NAP1L1, followed by co-staining for sialic acids/glycoproteins (in cell membranes/granular content) and the actin cytoskeleton using WGA or phalloidin, respectively.

Freshly isolated PLTs were fixed in suspension using PFA, permeabilized with 0.1% Triton X-100 (Thermo Fisher Scientific, AC327372500) for 5 min at room temperature (RT) and subsequently layered onto VECTABOND^TM^ (Vector Laboratories, Burlingame, CA, USA, SP1800) coated coverslips using a cytospin centrifuge (Shandon Cytospin, Thermo Fisher Scientific). Cells were incubated with IgG (to control for specificity of signal) or an antibody against NAP1L1 overnight at 4 °C. Cells were subsequently incubated with secondary antibodies conjugated to Alexa Fluor^®^ 488 or 546. Sialic acids/glycoproteins (in cell membranes/granular content) were co-stained using WGA.

### 4.9. Microscopy and Image Analysis

Fluorescence microscopy and high-resolution confocal reflection microscopy were performed using an Olympus IX81, FV300 (Olympus, Melville, NY) confocal-scanning microscope equipped with a 60×/1.42 NA oil objective for viewing PLTs. An Olympus FVS-PSU/IX2-UCB camera and scanning unit and Olympus Fluoview FV 300 image acquisition software version 5.0 were used for recording. In addition, an EVOS FL Auto Cell imaging system with an integrated dual camera system, system-specific software and equipped with a 609/1.42 NA oil objective was used. Monochrome 16-bit images were further analyzed, and changes were quantified using Adobe Photoshop CS6, ImageJ (NIH), and CellProfiler (www.cellprofiler.org (last accessed on 1 October 2022)) [96]. Analysis of images presented in Figure 5B was performed with a customized CellProfiler analysis set. In brief, raw images were automatically analyzed to minimize observer bias. The following software parameters were introduced: threshold method—otsu (this approach calculates the threshold separating three classes of pixels by minimizing the variance within each class); threshold correction factor—2.5 (a value > 1 makes the threshold more stringent, since the otsu method will give a slightly biased threshold if a larger percentage of the image is covered with positive signal).

### 4.10. Protein Expression Studies

All samples were normalized for starting cell concentrations. PLTs were lysed in Laemmli-buffer, and samples were separated by SDS-polyacrylamide gel electrophoresis (SDS-PAGE) and examined by western analysis for NAP1L1 or PDC-E2/ODP2 expression patterns. Subsequently, proteins were detected by enhanced chemiluminescence (ECL; Thermo Fisher Scientific, 32132). Linear range of detection was assured by using the saturation detection mode of the myECL Imager (Thermo Fisher Scientific, Waltham, MA, USA). For select studies a peptide competition assay was performed. In brief, a PVDF membrane loaded equally was split in half, and probed using anti-NAP1L1 antibodies pre-incubated with or without specific NAP1L1 peptide fragment (blocking peptide; incubated for 1 h at 37 °C in blocking buffer (TBS, Tween, non-fat dry milk)). Specific protein expression was normalized using ACTB/actin or b-tubulin expression and quantified using ImageJ (NIH).

### 4.11. Co-Immunoprecipitation Studies

For all modified protein co-IP studies, the Universal Magnetic Co-IP Kit (Active Motif, Carlsbad, CA, USA, 54002) was used according to the manufacturer’s protocol; however, magnetic beads were replaced using protein G magnetic beads specifically suited for immunoprecipitation (Dynabeads; Thermo Fisher Scientific, 10003D). In brief, 2 × 10^9^ total PLTs, or MKs were first pelleted and washed in PBS supplemented with phosphatase and deacetylase inhibitors. Cells were then lysed in 300 µL of the provided whole-cell lysis buffer and an additional protease inhibitor cocktail supplied by the manufacturer. The magnetic beads used for the IP reaction were either incubated with the antibody of interest (anti-NAP1L1) or the respective IgG control for 30 min at RT. Co-IP was performed using the prepared beads, the supplied buffer and the PLT cell lysate from 0.6 × 10^9^ PLTs or MKs with constant rotation for 4 h at 4 °C. The IP complexes were isolated and cleaned by placing the samples on a magnetic stand and performing repeated washes with the provided wash-buffer. Finally, each bead pellet was resuspended in 20 µL of Laemmli-buffer and heated to 99 °C for 3 min before being separated using SDS-PAGE or Western blot techniques, as described above. For mass spectrometry purposes, gels were directly stained using standard MS-compatible silver staining.

### 4.12. Mass Spectrometry

Protein bands of interest were manually picked using standard techniques to ensure minimal contamination with human skin particles. Excised protein bands were de-stained, extracted proteins were trypsin digested per established standard methods, and analyzed by an LTQ Orbitrap Velos MS coupled with a nano-acquity UPLC system (Thermo Fisher Scientific, and Waters Corporation, Milford, MA, USA) mass spectrometer with electro-spray ionization at the University of Greifswald at the Institute of Functional Genome Research. Repeat MS/MS fragmentation was controlled by applying dynamic exclusion using one repeat count, a repeat duration of 30 s, an exclusion list size of 500, and an exclusion duration of 60 s. Peptides were identified using SEQUEST [97], and analyzed using Scaffold (v.27 r.11, Proteome Software, Portland OR, USA) and the peptide/protein prophet algorithm [98], which was linked to the Uni-PROT/SWISS-PROT (human_v2014_02) data base, resulting in total spectrum counts per identified protein. Analysis settings included a peptide mass tolerance of 5 ppm, fragment ion tolerance of 0.5 Da, monoisotopic masses and trypsin cleavage (maximum two missed cleavages). Protein identifications were accepted when *p* < 0.05 after adjustment using the Scaffold mass correction, resulting in a false discovery rate of <1%.

### 4.13. PDH Activity Assay

All samples prepared and used for the performance of the PDH activity assay were normalized by cell number. The assay was conducted following the manufacturer’s protocol (abcam, ab109882). In brief, 50 µL of each sample was used and an equal volume of blocking fluid was added to a 96-well plate. The dipstick was dipped into the well and the sample allowed to wick onto the dipstick for 60 min. The dipstick was washed, and an activity buffer was added for 20 min. Since the activity reaction is an endpoint reaction, each dipstick was exposed for precisely monitored equal exposure times. The reaction was stopped using ddH_2_O. The results were documented using imaging scan techniques, and the signal was quantified using ImageJ (NIH).

### 4.14. NAP1L1 In Vitro Transcription

NAP1L1 was in vitro transcribed using a commercially available NAP1L1 expression plasmid (Origene, Rockville, MD, USA, RC201851) containing a T7 promotor for seamless in vitro transcription reactions. The linearized plasmid template, using the Pme I restriction site, was introduced into the in vitro transcription reaction (Thermo Fisher Scientific, AM 1345). The selected in vitro transcription kit, mMESSAGE mMACHINE™ T7 ULTRA Transcription Kit, was optimized to synthesize large amounts of efficiently and correctly capped mRNA, including a final poly(A) tailing step. The final mRNA product was subsequently isolated and purified using the MEGAclear kit (Thermo Fisher Scientific, AM 1908), and stored at −80 °C until used for transfection reactions.

### 4.15. NAP1L1 Transfection and Overexpression

CD34^+^-derived MKs were transfected on day 12 of the culture and were placed on immobilized fibrinogen on day 14 to induce proplatelet formation. Cells were transfected using Lipofectamine™ MessengerMAX™ Transfection Reagent per the manufacturer’s standard protocol. In brief, MKs on day 12 of the culture and differentiation course were used for the experiment. MesengerMAX reagent was diluted in Opti-MEM Medium (1:16.6). Next, mRNA master mixes were prepared by adding 1 µg of in vitro transcribed capped and poly(A)-tailed NAP1L1 mRNA, or its vehicle to 25 µL Opti-MEM Medium. The diluted mRNA was subsequently added to the dilute MessengerMAX, as prepared above, and incubated for 5 min. at room temperature. Transfection mixes were added to cells and incubated over night at 37 °C and 5% CO_2_. Cells were treated overnight with LPS 100 ng/mL or its vehicle, lysed in Laemmli-buffer, and samples were separated by SDS-polyacrylamide gel electrophoresis (SDS-PAGE) and examined by western analysis for NAP1L1 over-expression patterns.

### 4.16. Proplatelet Formation Assay

On day 13, the proplatelet forming (PPF) MK, defined as displaying at least one filamentous pseudopod, were scored by microscopy blinded as to experimental group, as previously described [17]. The percentage of PPF MK was calculated as the number of PPF MK compared to the total number of round cultured cells analyzed. An average of 200 cells were counted per condition.

### 4.17. Statistical Analyses

The mean ± SEM was determined for each variable. Student *t*-tests or ANOVA (where multiple groups were compared) was used to identify differences among two or multiple experimental groups, respectively (GraphPad Prism v9.1.0). If significant differences were found, Tukey’s multiple comparison test was used as recommended to determine the location of the difference (applicable for ANOVA). Basic science data were also examined for normality using Shapiro–Wilk and Kolmogorov–Smirnov tests. Non-parametric distribution was detected for the data presented in Figure 5E, and data was analyzed using Mann–Whitney-U/Wilcoxon Signed Rank test. For all analyses, *p*-value < 0.05 was considered significant, and exact *p*-values are indicated for each figure.

## Supplementary Materials

The following supporting information can be downloaded at: https://www.mdpi.com/article/10.3390/ijms232314694/s1, Figure S1: PLT activation does not change NAP1L1 expression pattern, Figure S2: MKs undergoing anaphase showed reduced NAP1L1 association with condensed chromosomes, Figure S3: NAP1L1-PDC-E2 interaction and pyruvate dehydrogenase complex activity are increased in MKs when exposed to agonist being present during sepsis.
