# Supplementary material for: A New Role of NAP1L1 in Megakaryocytes and Human Platelets
Source: Int J Mol Sci. 2022 Nov 24;23(23):14694. doi: 10.3390/ijms232314694 (PMC9737020; doi:10.3390/ijms232314694)
Supplement: Supplementary file 1 [file ijms-23-14694-s001.zip › ijms-1996450-supplementary.pdf]

Supplemental Figure S1. PLT activation does not change NAP1L1 expression pattern. RNA from freshly isolated PLTs at baseline (BL) and after incubation on immobilized fibrinogen (Fib) and stimulated with thrombin (Thr) was isolated and *NAP1L1* mRNA expression was evaluated by PCR. RT reaction omitting the enzyme are indicated by -RT. In addition, DNase treated samples are shown, and the -PCR lane demonstrates the negative control for the PCR reaction. Lanes labeled with M are indicating the marker lane. This figure is representative of 3 independent experiments. PCR-products are highlighted by yellow arrows.

Supplemental Figure S2. MKs undergoing anaphase showed reduced NAP1L1 association with condensed chromosomes. CD34+-derived MKs were fixed in suspension, spun on coverslips, and then incubated with an antibody against NAP1L1 (yellow, top right panel) or an IgG control (top left), followed by co-staining for actin (phalloidin, green), sialic acids (WGA, magenta), and nuclei (40,6-diamidino-2-phenylindole, DAPI, blue), as shown in the bottom panels. Scale bars = 20 µm. This figure is representative of n=7 independent experiments.

Supplemental Figure S3. NAP1L1 - PDC-E2 interaction and pyruvate dehydrogenase complex activity are increased in MKs when exposed to agonist being present during sepsis. (A) Bar graph showing the fold increase for LPS treated MKs over non-treated MKs of PDC-E2 interacting with NAP1L1. Grey bar represents normalized PDC-E2 co-IP

results for non-treated cells; non-filled bar shows increase for PDC-E2 detected after co-IP using LPS-treated cells. This figure is representative of n=6 independent experiments. **(B)** Pyruvate dehydrogenase complex activity assay was performed on MKs (day 13) untreated (noTx) or exposed to LPS. Plot depicts mean optical signal±SEM. This figure is representative of n=3 independent experiments.
