# Supplementary figures and images for: A New Role of NAP1L1 in Megakaryocytes and Human Platelets
Source: Int J Mol Sci. 2022 Nov 24;23(23):14694. doi: 10.3390/ijms232314694 (PMC9737020; doi:10.3390/ijms232314694)

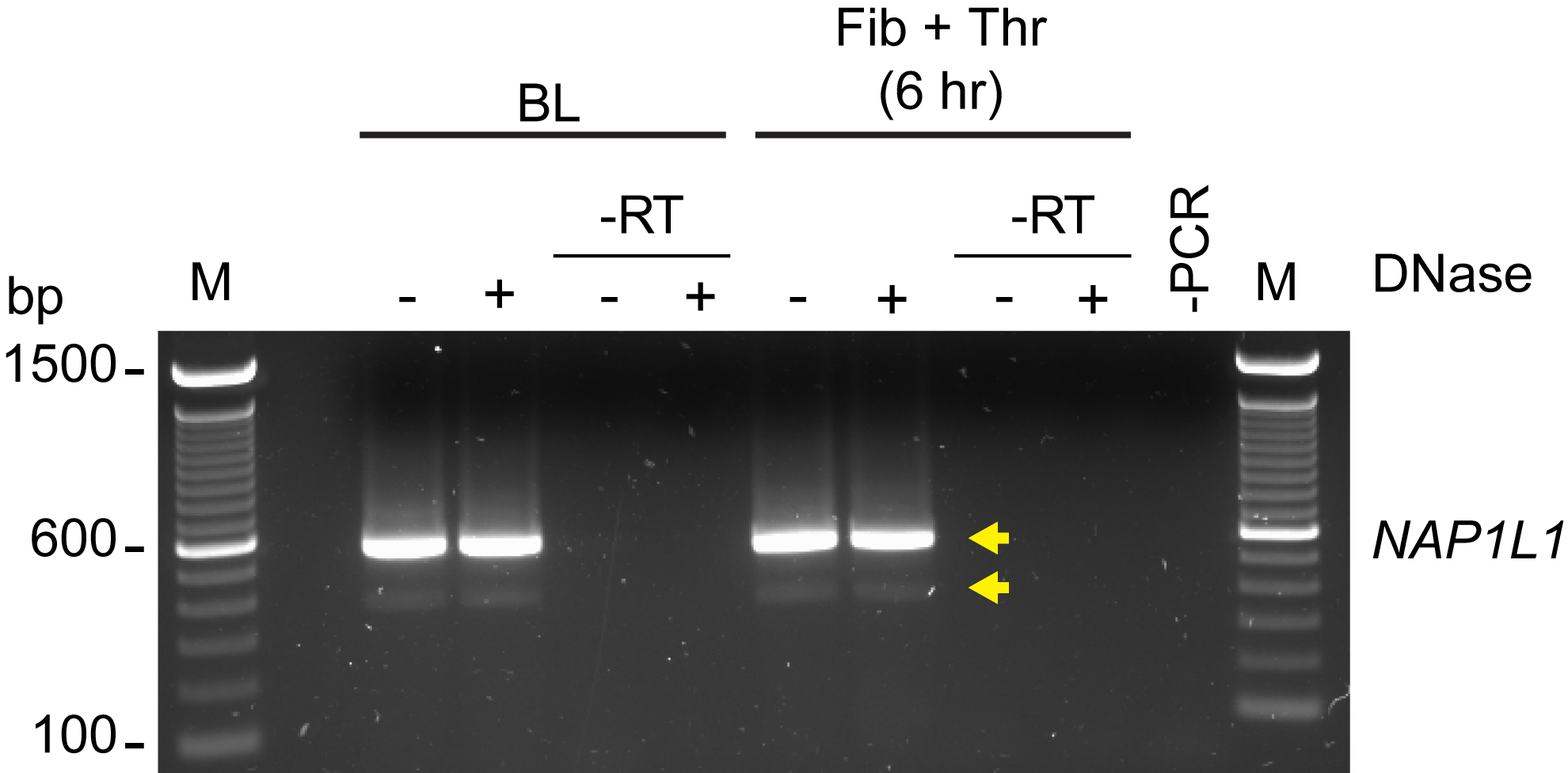

Supplement: Supplementary file 1 [file ijms-23-14694-s001.zip › Figure S1.tif]

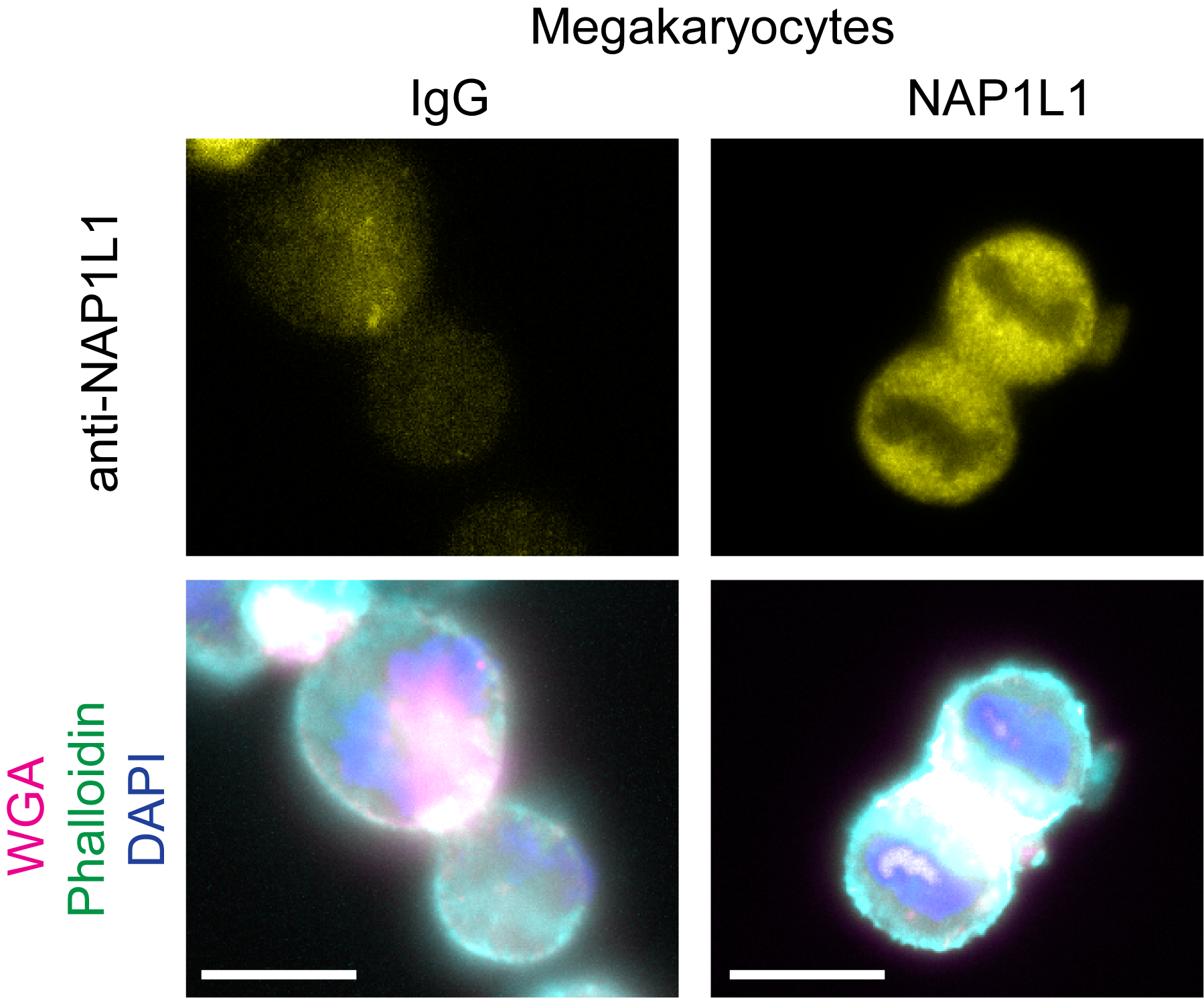

Supplement: Supplementary file 1 [file ijms-23-14694-s001.zip › Figure S2.tif]

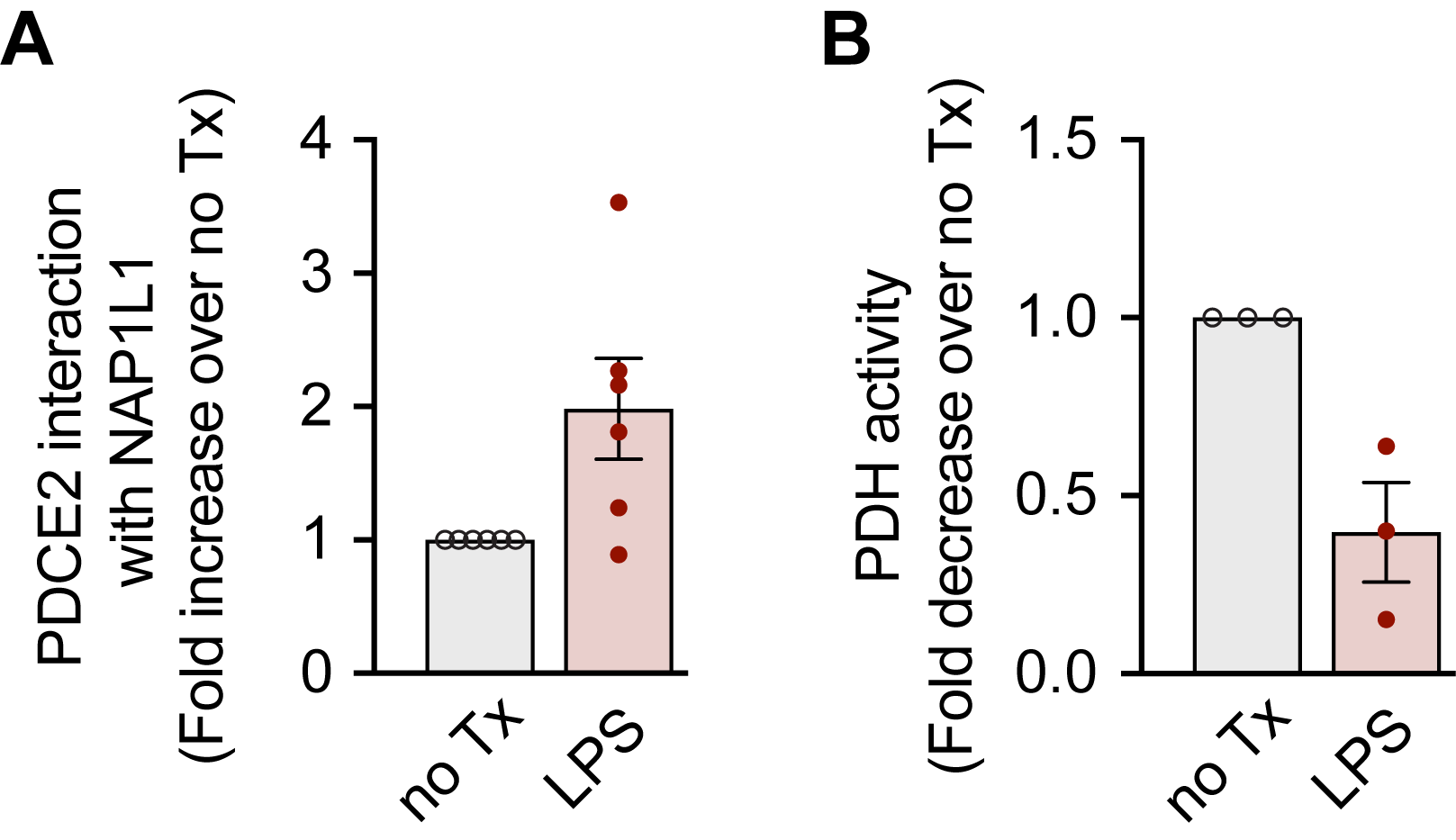

Supplement: Supplementary file 1 [file ijms-23-14694-s001.zip › Figure S3.tif]
